# Enhanced Combustion of Bituminous Coal and Semicoke Mixture by Ferric Oxide with Thermographic and Kinetic Analyses

**DOI:** 10.3390/ma14247696

**Published:** 2021-12-13

**Authors:** Tingting Lv, Luyao Kou, Tu Hu, Libo Zhang, Li Yang

**Affiliations:** 1 Faculty of Metallurgical and Energy Engineering, Kunming University of Science and Technology, Kunming 650093, China; lvtingting@stu.kust.edu.cn (T.L.); kouyao593858@163.com (L.K.); yanglikmust@163.com (L.Y.); 2 Yunnan Provincial Key Laboratory of Intensification Metallurgy, Kunming University of Science and Technology, Kunming 650093, China; 3 State Key Laboratory of Complex Nonferrous Metal Resources Clean Utilization, Kunming University of Science and Technology, Kunming 650093, China

**Keywords:** bituminous coal, semicoke, mixed coal powder, catalyst, thermographic and kinetic analysis

## Abstract

We study the specific catalytic effect of the catalyst on the combustion process of pulverized coal of increasing the proportion of semicoke in the mixture of semicoke and bituminous coal, and reducing the cost of blast-furnace coal injection. A combination of thermogravimetric and kinetic analyses were used to study the catalytic effect of Fe_2_O_3_ on semicoke and bituminous coal, and to improve the amount of semicoke in the mixed coal powder of bituminous coal and semicoke. Experimental results showed that Fe_2_O_3_ had a catalytic effect on both semicoke and bituminous coal, but there were differences in the catalytic stages of the same catalyst for different pulverized coal types. The addition of 2 wt % Fe_2_O_3_ to semicoke and bituminous coal each led to the ignition temperature and maximal burning rate temperature of the semicoke decreasing, indicating that the catalyst promoted the precipitation of a volatile fraction from the semicoke. The maximal burning rate temperature and burnout temperature of the bituminous coal decreased, and maximal weight loss rate increased, indicating that the catalyst promoted the combustion of the fixed carbon of bituminous coal. The optimal proportioning amount of semicoke in the mixed coal powder without the addition of a catalyst was 25%. After adding 2 wt % Fe_2_O_3_, the proportional amount of semicoke increased by 10%. The addition of the catalyst resulted in even lower activation energy for the same conversion rate. When the conversion rate was in the ranges of 0.1–0.2 and 0.5–0.7, the activation energy decreased by 22% and 26%, respectively, compared with that without a catalyst. Fe_2_O_3_ promoted the combustion of bituminous coal and semicoke. This enhanced the combustion performance of the pulverized coal mix and increased the proportion of semicoke in the mix, which has certain guiding significance in reducing the cost of blast-furnace iron making.

## 1. Introduction

Blast-furnace-pulverized coal injection is mainly a comingling of anthracite coals and bituminous coals. However, the reserves of anthracite coal in China and the amount that can be imported are gradually decreasing, resulting in the price of anthracite coal continuing to climb. So, to solve the problem of the increasing scarcity of anthracite coal, replacement coal powder for anthracite coal is sought. Semicoke, a new type of fuel, is produced from low-grade coal. Semicoke has a high fixed carbon content, and low-impurity S and P content. In China, reserves of low-rank coal are more than 50%, nearly 56 billion tons [1]. The use of semicoke for replacing anthracite coal is a good and suitable approach for China [2].

Semicoke is obtained by the low-temperature dry distillation of low-rank coal. In this process, carbon content increases as temperature increases, which is mainly because carbon releases small-molecular-weight gases during the combustion process [3]. The pore structure characteristics of semicoke may lead to unique combustion characteristics in comparison with those of other coal powders [2]. The low volatile fraction and low porosity of semicoke during pyrolysis are not conducive to ignition and combustion [4]. For semicoke to be available for efficient use, it is burned by preheating and mixed combustion [5,6]. However, the cost of preheating semicoke is high, and mixed combustion is mostly used. Mixed combustion means that the semicoke is burned with fuel with high volatile content. Some scholars studied the cocombustion properties of semicoke and biomass [4,7,8,9,10,11,12,13,14]. The mixed combustion of semicoke and pulverized coal has received more attention. Many scholars have studied the combustion characteristics of pulverized coal mixed with semicoke from different directions. Several studies showed that, as oxygen concentration increases, the burning characteristics of mixed coal powder improve, ignition and burnout temperatures decrease, and the comprehensive combustion characteristics index increases [15,16]. Yao et al. [2] found that activation energy increases with increasing oxygen content when semicoke and bituminous coal are burned in different O_2_/CO_2_ atmospheric ratios. Sun et al. [17] showed that, with increased heating rate, the ignition temperature of the pulverized coal mixture increased, the reaction was delayed, and the comprehensive combustion characteristic index increased, showing that the combustion performance of pulverized coal was improved. In addition to this, some studies have found the presence of catalysts in the combusted ash of pulverized coal to promote combustion, and some scholars have researched the influence of catalysts on the combustion performance of pulverized coal. Gopalakrishnan R et al. [18] found that the combustion rate of pulverized coal increased significantly when catalytic inorganic minerals in pulverized coal had been thoroughly mixed with pulverized coal. Hu et al. [19] studied the effect of catalysts and compared the promotion effect of different catalysts on pulverized coal combustion, and found that Fe_2_O_3_ had the best catalytic effect. Ma et al. [20] investigated the catalytic effect of three metal oxides (Fe_2_O_3_, MnO_2_, BaCO_3_) on pulverized coal combustion using thermogravimetric analysis (TGA). Results indicated that the three additives could enhance the combustion performance of coal powder, but there were differences in catalytic effect. Gong X et al. [21] studied the catalytic influence of CeO_2_ and Fe_2_O_3_ on the combustion of pulverized coal, and results indicated that these two catalysts improved the combustion performance of pulverized coal, and made the combustion of pulverized coal more complete.

The combustion of semicoke mixed with high volatile coal powder solves the problem of higher cost due to the scarcity of anthracite coal, and also improves the combustion performance of semicoke. However, in a mixture of semicoke and bituminous coal, the content of semicoke is limited. The results of Jiang et al. showed that Fushun oil shale (FS) in a mixture of Qinghai coal (QH) and Fushun oil shale (FS) had the largest ignition and burnout indices at 10% of the mixture [22]. The results of Zhang et al. showed that the contribution to burnout was stronger when the bituminous coal was 80% of the mixture of semicoke and bituminous coal [23]. It is an important goal to further increase the proportion of semicoke in the pulverized coal mixture and reduce the smelting cost without affecting the condition of the blast furnace. Therefore, it is necessary to study the influence of catalysts on the mixed combustion of semicoke and bituminous coal. Although many scholars believe that the catalyst has an improved effect on the combustion performance of pulverized coal, it is not clear exactly which part of pulverized coal combustion is catalyzed. It is also unclear how much the addition of a catalyst improves the proportional amount of semicoke in the mixture of semicoke and bituminous coal. Therefore, this paper explores the effect of catalysts on various stages of pulverized coal combustion, and finds the specific stages of pulverized coal combustion affected by catalysts. The amount of the improved proportion of semicoke in the coblend was obtained on the basis of the catalytic effect of the catalyst on the pulverized coal.

The mixed coal powder was prepared by mixing semicoke and bituminous coal according to the design ratio. The used catalyst was Fe_2_O_3_, which does not adversely interfere with the blast-furnace iron-making process, and is cheap and easily available. First, the optimal ratio of semicoke instead of anthracite coal mixed with bituminous coal for combustion was analyzed (the comprehensive combustion characteristics index of bituminous coal:anthracite coal = 1:1 was used as the standard for comparison). Then, the catalytic effect of the catalyst on the combustion of semicoke and bituminous coal was analyzed by the change in the combustion characteristics parameters of pulverized coal. Then, the change in the amount of semicoke ration in the mixed pulverized coal after adding the catalyst was analyzed by combustion characteristic parameters. Lastly, the effect of the catalyst on the activation energy of mixed pulverized coal was studied with kinetics. The adopted method was adding a Fe_2_O_3_ catalyst to the mixed coal powder to increase the semicoke proportioning amount, which is important for blast-furnace iron making, mainly to improve blast-furnace production efficiency and reduce iron-making costs.

## 2. Materials and Methods

### 2.1. Raw Materials

The anthracite, bituminous coal, and semicoke used in the experiments were all from a Sichuan steel company (Pangang Group Co. LTD, Panzhihua, China) to avoid the effects of moisture in the pulverized coal. The three types of pulverized coal were dried in a constant-temperature oven (Heng Scientific Instruments Ltd., Shanghai, China) at 105 °C for 3 h, crushed and ground with a crusher (Wuhan Prospecting Machinery Factory, Wuhan, China), sieved in a 200 mesh, and stored in a sealed bag for backup. The compositional analysis of the three coal powders from a steel company in Sichuan is shown in Table 1, which shows that the ash, volatile, and sulfur contents of the three pulverized coals met the indices required for blast-furnace blowing. The fixed carbon content of semicoke was the highest, 85.85%, and the ash and sulfur content of semicoke were the lowest, 8.78% and 0.22%, respectively. The catalyst used in this study was analytical-grade pure Fe_2_O_3_. Samples were mixed according to the designed proportions. The mixing ratios of the semicoke for this study were 15%, 20%, 25%, 30%, 35%, 40%, and 50%. The mixture of anthracite coal was 50%. We added 1, 2, and 3 wt % of Fe_2_O_3_ to the specified coal powder and uniformly mixed it.

### 2.2. Thermogravimetric Analysis

**Figure 1:**
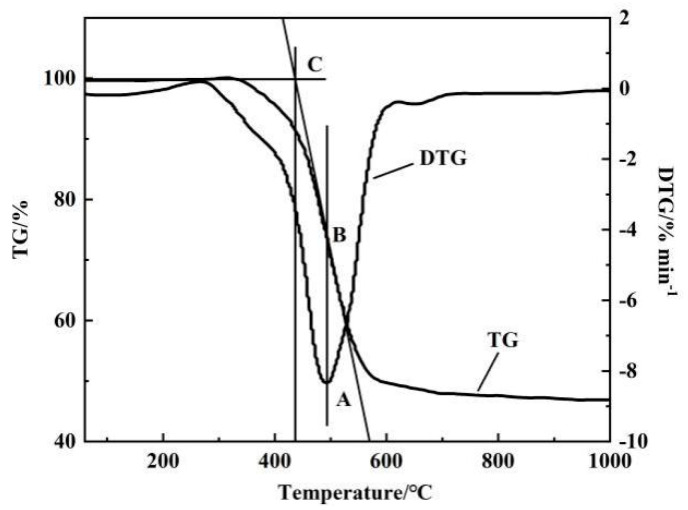
TG–DTG curve [25,26,27].

This experiment investigates the reaction characteristics of pulverized coal combustion by means of TG(Thermogravimetry) and DTG (Derivative Thermogravimetry) curves. Thermogravimetric experiments were carried out using a thermogravimetric analyzer (NETZSCH STA 2500, NETZSCH, Bavaria, Germany). The mass of each experimental sample was 6 ± 0.1 mg, and test samples were placed in an aluminum crucible. The experimental atmosphere was air, and the gas flow rate was 60 mL/min. The experimental temperature was increased from room temperature to 1100 °C at different heating rates (10, 20, 30, and 40° C/min). The combustion characteristic parameters of pulverized coal were mainly obtained from the TG and DTG curves, and calculations. In this experiment, the burning performance of pulverized coal was judged by comparing variations in the characteristic parameters of pulverized coal combustion. Figure 1 shows the TG–DTG curve graph.

From the TG–DTG tangent method [24], the ignition, burnout, and maximal burning-rate temperatures of the samples could be determined. The ignition temperature is shown in Figure 1 [25,26,27]. First, a plumb line was drawn from the peak point of DTG curve that intersected the TG curve at point B. A tangent line was then drawn through point B, intersecting with the horizontal tangent line of TG curve at point C. The horizontal coordinate of point C is ignition temperature T_i_. The horizontal coordinate of point A is T_max_.

The temperature at which the combustion process lost more than 98% of its weight is burnout temperature T_b_ [28]. Comprehensive combustion characteristic index S is determined by Equation (1). The higher the S value is, the better the combustion performance of the pulverized coal.

(1) $$S=\frac{{\left(\mathrm{dw}/\mathrm{dt}\right)}_{\max}\times {\left(\mathrm{dw}/\mathrm{dt}\right)}_{\mathrm{mean}}}{T_{i}^{2}T_{b}}$$

where ${\left(\mathrm{dw}/\mathrm{dt}\right)}_{\max}$ is the maximal burning rate of the sample (%/min); ${\left(\mathrm{dw}/\mathrm{dt}\right)}_{\mathrm{mean}}$ is the average burning rate of the sample (%/min); T_i_ is the ignition temperature of the sample (°C); T_b_ is the combustion temperature of the sample (°C). The average burning rate was calculated by the following equation.

(2) $${\left(\mathrm{dw}/\mathrm{dt}\right)}_{\mathrm{mean}}=\beta \times \frac{\alpha_{i}-\alpha_{b}}{T_{b}-T_{I}}$$

where β is the heating rate of the thermal analyzer (°C/min); $\alpha_{i}$ is the mass fraction of the sample when it catches fire (%); and $\alpha_{b}$ is the mass fraction of the sample when it burns out (%).

### 2.3. Kinetic Analysis

The isoconversion method is also known as the model-free method, which means that the kinetic parameters of the reaction can still be calculated more accurately in the absence of the mechanism function. The mechanism function does not change with the heating rate at the same conversion rate [6]. The combustion of a mixture of semicoke and bituminous coal is a complex gas–solid phase reaction, and the reaction is affected by temperature, atmosphere, and the rate of temperature rise [29], so the isoconversion method is more suitable for solving the activation energy. There are various isoconversion methods, and the Flynn–Wall–Ozawa (FWO) method [30,31] was chosen here to calculate the activation. Equation (3) is the kinetic equation for the nonisothermal gas–solid phase reaction.

(3) $$\frac{d\alpha }{\mathrm{dt}}=A\;\exp\left(-\frac{E}{\mathrm{RT}}\right)\times f\left(\alpha \right)$$

where α is the conversion (%); A is the prefactor; apparent activation energy is E (kJ/mol); the mechanism function is f(α); T is the temperature, K, corresponding to the conversion rate α; the time corresponding to the conversion rate α is t; and gas constant R = 8.31447 J/molK.

Heating rate in Equation (3) is calculated as follows.

(4) $$\frac{d\alpha }{\mathrm{dT}}=A\;\exp\left(-\frac{E}{\mathrm{RT}}\right)\times f\left(\alpha \right)$$

(5) $$\alpha =\frac{m_{i}-m_{t}}{m_{i}-m_{\infty }}$$

where α is calculated by formula, Mm_i_ and m_∞_ are the initial and final weights of the samples, respectively; m_t_ is the weight at moment t.

The integral deformation of Equation (4) leads to the kinetic model function corresponding to the Flynn–Wall–Ozawa (FWO) method, as shown in Equation (6).

(6) $$\ln\beta =\ln\frac{0.0048\mathrm{AE}}{\mathrm{Rg}\left(\alpha \right)}-1.0516\frac{E}{\mathrm{RT}}$$

(7) $$g\left(\alpha \right)=\int_{0}^{\alpha }\frac{1}{f\left(\alpha \right)}d\beta$$

A different heating rate β, the first term on the right-hand side of Equation (6) is constant for the same conversion rate α [15]. From the graph of lnβ-1/T, each conversion rate in the test had an activation energy (slope of the curve) corresponding to it.

## 3. Results

### 3.1. Thermal Analysis of Mixed Combustion of Semicoke and Bituminous Coal

**Figure 2:**
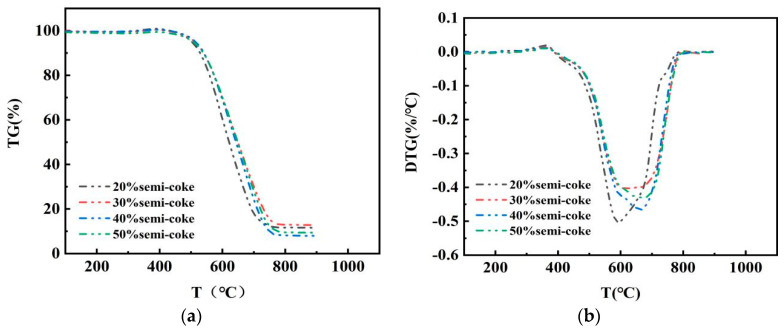
TG–DTG for combustion of semicoke mixed with bituminous coal with different addition ratios. (**a**) TG and (**b**) DTG curves for different semicoke ratios in pulverized coal blends.

The mixing ratio of pulverized coal met the In this study, comprehensive combustion characteristic index S of the current blended pulverized coal ratio (bituminous coal:anthracite coal = 1:1) of a smelter in Sichuan was used as the reference standard, as shown in Table 2. If S > S standard for the mixed pulverized coal of bituminous coal and semicoke, this indicated that requirement; otherwise, it did not meet. In order to explore the amount of semicoke and bituminous coal ratios that meet blast-furnace-pulverized coal injection requirements, semicoke and bituminous coal were mixed and burned at different mass ratios (2:8, 3:7, 4:6, 1:1) at a heating rate of 20 °C/min. The optimal mixing ratio of semicoke and bituminous coal was found. Figure 2 shows the TG–DTG curve for the combustion process of pulverized coal with different mixing ratios. 

**Figure 3:**
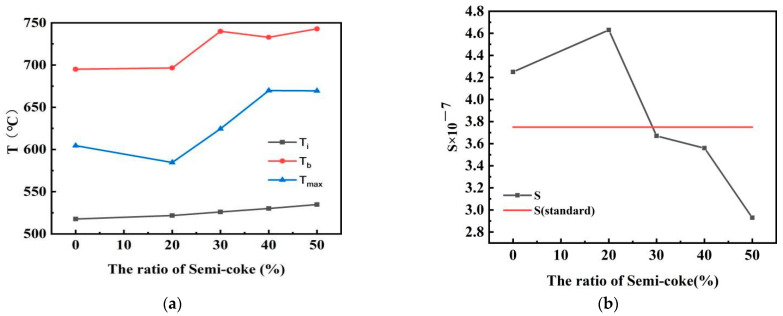
Combustion characteristics parameters of the mixture of semicoke and bituminous coal with different addition ratios: (**a**) Ti, Tb, Tmax, and (**b**) S for different semicoke ratios in mixed pulverized coal.

The combustion of pulverized coal after drying goes through two main stages [32]: the combustion (1) of the volatile fraction and (2) of fixed carbon. This is different from the coal that has not been directly dried and burned [33]. Figure 2 shows that TG curves are smooth in the weight-loss phase, indicating that the combustion of the volatile fraction and fixed carbon are closely linked during the combustion process. The general trend of the TG curve is similar regardless of the amount of dispensed semicoke. With the increase in the proportion of semicoke, the DTG curve shifts backward, and the maximal weight-loss rate decreases. Combining TG–DTG curves with calculations, combustion characteristic parameter curves were obtained for different ratios of semicoke and bituminous coal, as shown in Figure 3. Figure 3 shows that, at a half-coke ratio of 20%, the ignition and burnout temperatures of the pulverized coal mixture at that time showed a decreasing trend, and the S of the mixed pulverized coal was larger than the value of either bituminous coal or semicoke burning alone. This means that the combustion of semicoke and bituminous coal was mutually reinforcing. When the proportion of semicoke in the pulverized coal mixture was less than 20%, the combustion performance of the pulverized coal mixture mainly depended on the combustion performance of the semicoke, so the combustion performance of the pulverized coal mixture gradually increased as the mass ratio of semicoke increased. When the mass ratio of semicoke in mixed pulverized coal is higher than 20%, the combustion performance of mixed pulverized coal is mainly determined by the combustion performance of bituminous coal, so the combustion performance of mixed pulverized coal gradually deteriorates when the mass ratio of semicoke increases [34]. When the semicoke ratio was 20%, comprehensive combustion characteristic index S was higher than the S standard and met the requirements. However, when the semicoke ratio reached 30%, comprehensive combustion characteristic index S was lower than the S standard, indicating that the amount of semicoke was too high and did not meet the requirements. This analysis shows that the optimal amount of semicoke ration was between 20% and 30%. To further determine the optimal mass ratio of semicoke, different mass ratios (15:85 and 25:75) of semicoke and bituminous coal were accurately weighed and uniformly mixed. The combustion characteristic parameters calculated from the TG–DTG curves are shown in Table 3, which indicates that, when the proportional amount of semicoke was 25%, comprehensive combustion characteristic parameter S was larger than S standard, which met the requirements. The above analysis shows that the optimal proportion of semicoke in the coal mixture is 25%.

### 3.2. Effect of Fe_2_O_3_ on Combustion of Semicoke and Bituminous Coal

**Figure 4:**
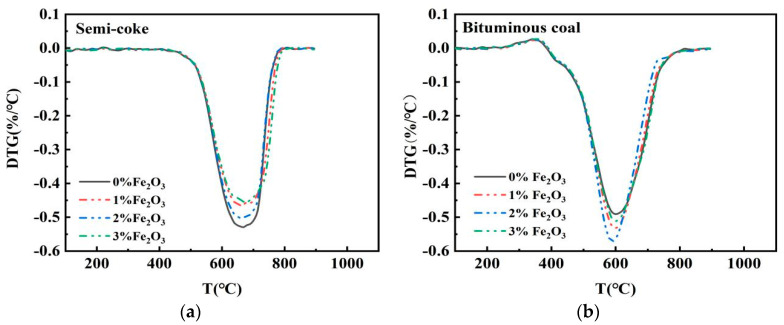
DTG curves for (**a**) semicoke and (**b**) bituminous coal at different addition levels of Fe_2_O_3_.

**Figure 5:**
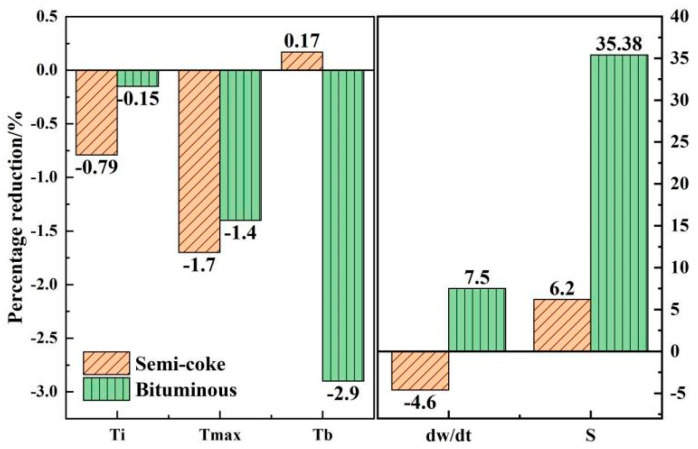
Variation of combustion characteristic parameters of semicoke and bituminous coal after adding catalyst.

It is necessary to further increase the proportional amount of semicoke and to study the catalytic effect of the catalyst on pulverized coal combustion. Combustion tests were carried out on semicoke and bituminous coal samples with Fe_2_O_3_ additions of 1, 2, and 3 wt %, using a thermal analyzer. The effect of Fe_2_O_3_ with different addition ratios on the combustion performance of and bituminous coal and semicoke was studied. Experimentally obtained DTG curves are shown in Figure 4, which indicates that both pulverized coal types showed the best catalytic effect with the addition of 2 wt % Fe_2_O_3_, and there was a difference in the catalytic effect of the catalysts for the two types of pulverized coal. The maximal combustion rate decreased with the addition of the catalyst to the semicoke. However, for bituminous coal, the addition of the catalyst significantly increased the maximal combustion rate. The peaks of the DTG curves narrowed at 2 wt % of catalyst addition, indicating a more concentrated combustion of bituminous coal. To further comprehend the difference in catalytic effect, the combustion characteristic parameters of semicoke and bituminous coal with 2 wt % Fe_2_O_3_ were selected for comparison. Combustion characteristic parameter curves are shown in Figure 5.

Figure 5 shows that, with the addition of 2 wt % Fe_2_O_3_ to semicoke, the ignition temperature and maximal combustion-rate temperature of semicoke both decreased by 0.79% and 1.7%, respectively, and the burnout temperature of semicoke increased by 0.17%. In contrast, when 2 wt % Fe_2_O_3_ was added to the bituminous coal, its ignition temperature was basically unchanged, its maximal combustion-rate temperature decreased by 1.4%, and its burnout temperature decreased by 2.9%. Figure 5 also shows that the maximal weight-loss rate of semicoke decreased by 4.6%, and the comprehensive combustion characteristic index increased by 6.2%; and the maximal combustion rate and comprehensive combustion characteristic index of bituminous coal increased by 7.5% and 35.38%, respectively. Comparing the different catalytic effects of catalysts on bituminous coal and semicoke showed that the addition of 2 wt % Fe_2_O_3_ had a greater effect on the early stages of the combustion phase of semicoke, and a greater effect on the middle and late stages of the combustion phase of bituminous coal. Fe_2_O_3_ mainly plays a role in transferring oxygen during the reaction. The same conclusion was obtained from their mechanistic diagrams [35,36]. As temperature gradually increased, a reducing atmosphere appeared around the pulverized coal that oxidized Fe_2_O_3_ into FeO [35], making the oxygen content around the pulverized coal more adequate and promoting the reaction. The level of volatile matter directly controls the pyrolysis process of pulverized coal [37]. The low volatile content of semicoke makes the pulverized coal less likely to catch fire. The results of Yao et al. showed that the ignition temperature of semicoke was higher than that of bituminous coal [2]. Adding catalysts to the semicoke increased the oxygen content, which increased the oxygen-containing functional groups and artificially lowered the degree of coal quality, facilitating the reaction [38]. At the same time, when the amount of oxygen was higher, the release of volatile fraction in the semicoke was more concentrated, the ignition temperature increased, and the maximal combustion-rate temperature was accordingly reduced. The volatile fraction of bituminous coal is high, so Fe_2_O_3_ has almost no effect on reducing the ignition temperature. However, as the combustion reaction proceeded, the temperature and pressure around the bituminous coal gradually increased, and the diffusion rate of oxygen reaching the surface of pulverized coal slowed down. The addition of the catalyst at that point increased the oxygen content around the pulverized coal, allowing for the bituminous coal to burn more quickly and completely. After the combustion of the volatile fraction of pulverized coal, only the combustion of fixed carbon remained, but the catalytic effect on the combustion of fixed carbon of semicoke and bituminous coal significantly differed. The main reason is the difference in the composition of the two pulverized coals. Compared to bituminous coal, semicoke has high fixed carbon content and low volatile content, which results in semicoke not being easily decomposed during combustion, and porosity being relatively low. The catalyst is not evenly distributed in the semicoke, so the catalytic effect is not as obvious as that of bituminous coal.

### 3.3. Study of Catalysts for Mixed Combustion of Bituminous Coal and Semicoke

The optimal amount of semicoke in the mixed pulverized coal was 25% when no Fe_2_O_3_ catalyst was added. When the semicoke share was 30%, the comprehensive combustion characteristic index of the mixed coal powder was lower than the standard value and did not meet the requirements of pulverized coal injection. The above analysis showed that the catalyst improved the combustion of pulverized coal. In order to improve the semicoke proportion, 2 wt % Fe_2_O_3_ was added to the mixed pulverized coal with 30%, 35%, and 40% semicoke proportions. The combustion characteristic parameters of the mixed pulverized coal were obtained and are shown in Table 4. After adding the catalyst, the semicoke proportioning amount reached 35%, which was improved by 10%. The addition of catalysts promoted the oxidation and conversion of nonoxidizable and nonconvertible substances in the pulverized coal [32]. In addition, the increase in oxygen content promoted the diffusion of oxygen, which promoted the precipitation of the volatile fraction of semicoke and the combustion of the fixed carbon of bituminous coal. This led to an increase in the maximal combustion rate of the pulverized coal mixture, a decrease in the burnout temperature, and an increase in the comprehensive combustion characteristic parameters. The combustion was more complete. The final result was an increase in the proportion of semicoke.

### 3.4. Effect of Temperature Rise Rate on the Combustion of Pulverized Coal Mixture

**Figure 6:**
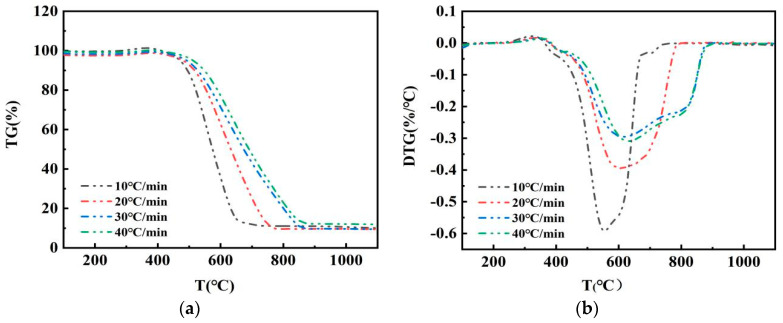
(**a**) TG and (**b**) DTG curves for different heating rates.

To investigate the effect of heating rate on the combustion of pulverized coal mixtures, pulverized coal with a semicoke ratio of 30% was heated in an air atmosphere with heating rates of 10, 20, 30, and 40 °C/min. Figure 6 shows that the combustion characteristic parameters of the pulverized coal blend differed for different heating rates, but the trend of the curves was the same, showing that the heating rate did not affect the combustion mechanism of pulverized coal. The maximal combustion rate of the DTG curve shifted to the right as the rate of warming increased, which means that the reactions were delayed. When the heating rate is high, heat conversion takes time, enlarging the temperature gradient inside and outside the fuel particles, which go against the precipitation of volatile components, ignition temperature increases, and the reaction shifts back [17]. After multiplying the peak point of DTG curve in Figure 6 with the corresponding heating rate, the maximal burning rate was the smallest when the temperature rise rate was 10 °C/min, and the maximal burning rate was the largest when the temperature rise rate was 40 °C/min. When the heating rate is low, the combustion process of pulverized coal is slow and takes a long time. When the heating rate is too high, the combustion time is short, and unburned powder increases, which is not conducive to the full combustion of pulverized coal. Taking this into account, the best heating rate was 20 °C/min.

### 3.5. Kinetic Analysis of Pulverised Coal Combustion

**Figure 7:**
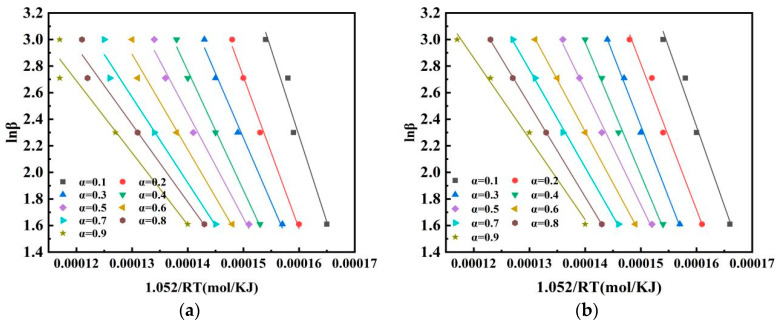
Fitting curves of activation energy (**a**) without and (**b**) with catalyst. Curve fitted by FWO method in the (**a**) absence and (**b**) presence of the catalyst.

**Figure 8:**
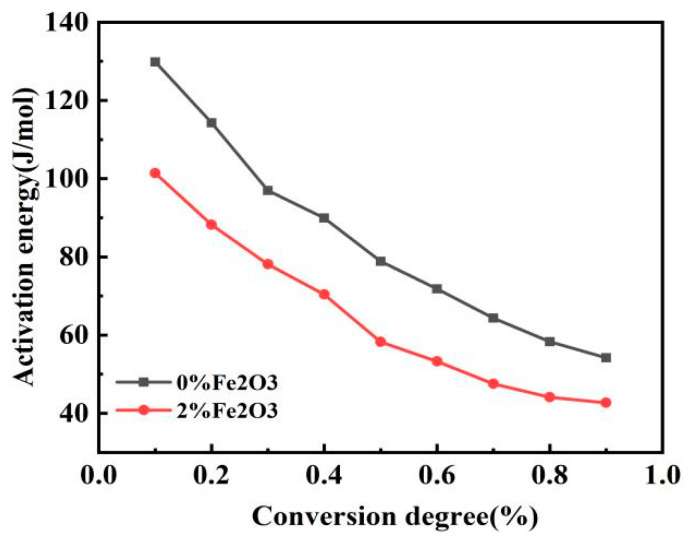
Graph of conversion rate.

**Figure 9:**
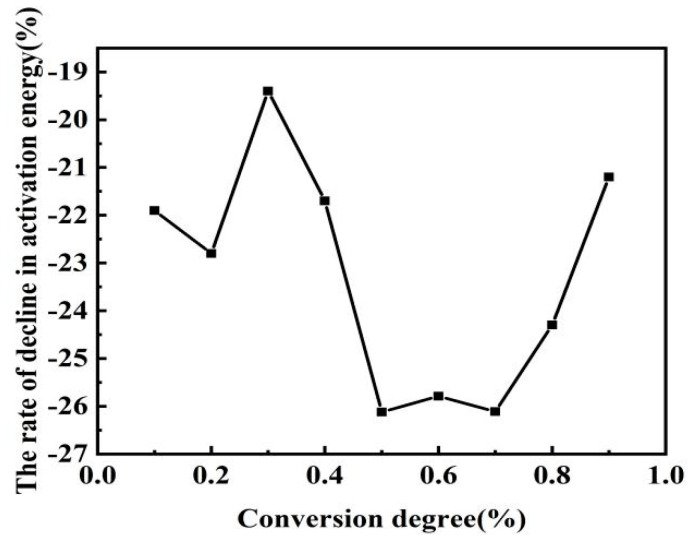
Rate of change in activation energy.

The isoconversion method is called the model-free method [2], with which kinetic parameters can more accurately calculate the reaction without a mechanistic function. The Flynn–Wall–Ozawa (FWO) method was used to evaluate the relationship between the mixed pulverized coal conversion rate and activation energy [30,31] according to different heating rates (10, 20, 30, and 40 °C/min). The lnβ vs 1/T curve fitted by the Flynn–Wall–Ozawa equation is shown in Figure 7. Wang et al. chose the method with a higher correlation coefficient when studying kinetics [4]. The closer the correlation coefficient of the fitted curve is to 1, the better the correlation of the curve is. On the basis of kinetic equations and fitted curves, the different conversion rates of activation energies can be calculated. In this study, activation energies were calculated for a conversion rate between 0.1 and 0.9, as shown in Table 5. The link between activation energy and percentage conversion in the table is shown in Figure 8, which indicates that the activation energy of the pulverized coal blend gradually decreased as the conversion rate increased, with or without the addition of the catalyst. Many scholars concluded that activation energy increases with conversion rate [15,21,27]. Pulverized coal combustion begins with the precipitation of the volatile fraction, followed immediately by the combustion of the volatile fraction, and lastly by the combustion of fixed carbon. The volatile fraction is burned before the fixed carbon is, so the fixed carbon is preheated before combustion [26], which helps to reduce the activation energy and facilitates the reaction. Comparing the two curves in the figure shows that the activation energy at the same conversion rate was lower after adding the catalyst. The activation energy at the same conversion rate was compared to obtain the variation in activation energy reduction for both coal powders, as shown in Figure 9. After adding the catalyst, the reduction in activation energy was relatively large in the two ranges of 0.1–0.2 and 0.5–0.7, namely, 22% and 26%, respectively. This means that the required energy for the reaction of the mixed coal powder in this stage was reduced. This further proves the previous point that catalysts catalyze different coal powders differently. When the conversion rate was 0.7, the reduction in activation energy rapidly decreased. At that time, the reaction reached the end of combustion, and there was ash wrapped around the mixed pulverized coal, which was not conducive to the combustion of pulverized coal. The ash covered the semicoke surface and obstructed the pores of the pulverized coal, which inhibited carbon and oxygen contact [29]. Although the addition of a catalyst can provide oxygen, ash also hinders the contact between oxygen and pulverized coal to a certain extent, and hinders the diffusion of reaction products. 

## 4. Conclusions

This paper focused on the catalytic effect of Fe_2_O_3_ on the combustion of semicoke and bituminous coal, and the effect of the catalyst on the proportional amount of semicoke in the coblending of bituminous coal and semicoke using thermogravimetric and kinetic analyses. Results showed that 2 wt % Fe_2_O_3_ had a catalytic effect on the combustion of both pulverized coal types, but there were huge difference in the stage of catalysis. The catalyst mainly affected the early stage of semicoke combustion, which led to the pyrolysis of the volatile fraction being more concentrated. For bituminous coal, it mainly affected the middle and late stages of bituminous combustion, leading to the combustion of fixed carbon being more complete. Since the catalyst improved the combustion performance of both semicoke and bituminous coal at different stages, the addition of the catalyst also increased the amount of semicoke in the coblend of bituminous coal and semicoke from 25% to 35%. The activation energy of the blended pulverized coal decreased less with the increase in conversion rate after the addition of the catalyst, and the conversion rate decreased more when it was in the ranges of 0.1–0.2 and 0.5–0.7. This further indicated that there were discrepancies in catalytic stages of catalysts for different pulverized coal types.

## Figures and Tables

**Table 1:** Composition analysis of three kinds of pulverized coal (wt %).

Coal Type	Proximate Analysis (wt %)				
	FC_ad_	M_ad_	A_ad_	V_ad_	St_ad_
Anthracite coal	79.76	0.27	11.48	8.76	0.60
Bituminous coal	75.76	0.37	10.78	13.09	0.52
Semicoke	85.85	0.64	8.78	4.73	0.22

**Table 2:** Combustion parameters for anthracite and bituminous coal after uniform mixing at 1:1.

Samples	T_i_/°C	T_b_/°C	T_max_/°C	(dw/dt)_max_/%·min^−1^	S/×10^−7^
50% anthracite coal	523.99	707.32	604.15	9.66	3.86

**Table 3:** Combustion characteristic parameters of the mixture of semicoke and bituminous coal with different addition ratios.

Samples	T_i_/°C	T_b_/°C	T_max_/°C	(dw/dt)_max_/%·min^−1^	S/×10^−7^
15% semicoke	524.77	704.56	599.71	10.00	4.24
25% semicoke	524.74	706.82	599.53	9.86	4.23

**Table 4:** Proportion of semicoke in a mixture of semicoke and bituminous pulverized coal.

Semicoke Ratio	T_i_/°C	T_b_/°C	T_max_/°C	(dm/dt)_max_/%·Min^−1^	S/×10^−7^
30%	524.82	712.46	594.28	9.6	4.09
35%	524.65	709.19	624.5	9.52	3.95
40%	521.73	711.83	589.62	9.02	3.52

**Table 5:** Link between activation energy and percentage conversion.

Conversion Rate	0 wt% Fe_2_O_3_		2 wt% Fe_2_O_3_	
	E	R^2^	E	R^2^
0.1	129.8	0.95822	101.4	0.96964
0.2	114.3	0.98655	88.1	0.97238
0.3	96.9	0.98757	78.1	0.99442
0.4	89.9	0.99106	70.4	0.98818
0.5	78.8	0.97982	58.2	0.99229
0.6	71.8	0.96669	53.2	0.99959
0.7	64.3	0.96475	47.5	0.99815
0.8	58.3	0.96298	44.1	0.99988
0.9	54.1	0.94195	42.6	0.99133
Average	84.24	0.971	64.84	0.98954

## Data Availability

The data in this study are available on request from the corresponding author.

## Abbreviations

FC_ad_	Fixed carbon
M_ad_	Moisture
A_ad_	Ash
V_ad_	Volatiles
St_ad_	Sulphur content
T_i_	Ignition temperature
T_max_	Maximal burning rate temperature
T_b_	Burnout temperature
ω	Mass percentage
S	Comprehensive combustion characteristic index
β	Heating rate
α_i_	Mass fraction of the sample when it catches fire
α_b_	Mass fraction of the sample when it burns out
t	Time
T	Temperature
A	Pre-exponential factor
E	Activation energy
R	Gas constant = 8.31447 J/molK
m_i_	Initial weights of the samples
m_t_	Weight at moment t
m_∞_	Final weights of the samples
R^2^	Correlation coefficient
